# The Efficacy of the Smartphone App for the Self-Management of Low Back Pain: A Systematic Review and Assessment of Their Quality through the Mobile Application Rating Scale (MARS) in Italy

**DOI:** 10.3390/life14060760

**Published:** 2024-06-13

**Authors:** Luca Scala, Gloria Giglioni, Luca Bertazzoni, Francesca Bonetti

**Affiliations:** 1 Department of Clinical Sciences and Translational Medicine, University of Rome Tor Vergata, 00133 Rome, Italy; gloria.giglioni1@gmail.com (G.G.); francesca.bonetti@uniroma2.it (F.B.); 2 Physioup—Physiotherapy Practice, 00142 Rome, Italy; 3 Asl Roma 3, Department of Rehabilitation, 00122 Rome, Italy; 4 Pain in Motion Research Group (PAIN), Vrije Universiteit, 1050 Brussels, Belgium; luca.bertazzoni@vub.be; 5 Painlab Studio Osteopatico, 20145 Milan, Italy

**Keywords:** digital health, mobile health unit, rehabilitation, musculoskeletal diseases

## Abstract

Smartphone apps for self-management are valuable tools to help manage low back pain (LBP) patients. The purposes of this systematic review were to (a) summarize the available studies on the efficacy of smartphone apps for self-management of LBP and (b) identify free applications available in Italy that offer strategies for LBP self-management and provide a qualitative assessment using the Mobile Application Rating Scale (MARS). According to the Prisma Checklist, six bibliographic databases were searched with the keywords ‘low back pain’, ‘mobile application’, ‘smartphone’, and ‘telemedicine’. In total, 852 records were screened, and 16 were included in the systematic review. Of the six RCTs included, four reported a statistically significant decrease in pain in favor of the app group, and two RCTs did not. Only in a non-RCT was there an increase in the disability score. In the application research conducted on mobile stores, we identified and rated 25 applications through MARS. The overall scores ranged from 1.93 to 3.92 for the IOS app and 1.73 to 4.25 for the Play Store app. The findings suggest that few apps meet satisfying quality, content, and functionality criteria for LBP self-management.

## 1. Introduction

Low back pain (LBP) is the musculoskeletal disorder with the most significant social burden and is considered the leading cause of disability worldwide [1]. Healthcare costs and disability related to low back pain vary substantially among countries and are influenced by cultural and social contexts, as well as beliefs about the condition [1,2]. The disability and costs associated with low back pain are projected to increase in the coming years, particularly in developing countries with limited economic resources [1,2].

Recent evidence suggests that self-management strategies are effective and should be promoted for patients with chronic low back pain [3,4]. During the COVID-19 pandemic, healthcare services have had to adapt to ensure the safe delivery of care, which has led to a reduction in outpatient services and an increased reliance on alternative methods for providing rehabilitation and physical therapy.

Smartphone apps are increasingly being used to help patients with chronic conditions [5,6,7]. Self-management interventions delivered via mobile apps are valuable tools for supporting the management of chronic LBP [5]. These apps help reduce costs and end geographical barriers, making rehabilitation services more accessible to all.

Mobile health (mHealth) apps for pain management offer potential benefits to patients by enabling the monitoring of acute or chronic pain and providing information and support for pain management [7].

However, there is limited literature exploring the effectiveness of smartphone apps for self-managing low back pain (LBP), and the current evidence is still inconclusive [8]. Furthermore, no rigorous evaluation of LBP self-management apps available in Italy has been conducted, and there is no guidance for healthcare professionals and consumers on selecting high-quality, evidence-based LBP apps.

Given the vast number of publicly available apps for LBP self-management, it is crucial to assess their quality and ensure they adhere to best practice guidelines.

The primary aim of this systematic review is to provide an updated synthesis of existing primary studies on the effectiveness of smartphone apps for self-managing LBP. Additionally, the secondary aim is to identify free apps available in Italy that offer strategies for LBP self-management and to provide a qualitative evaluation using the Mobile Application Rating Scale (MARS) [9] to clarify the validity of the content provided to users.

## 2. Materials and Methods

Our systematic review, registered with PROSPERO (ID: CRD42022357170), employs a mixed-methods approach to provide an overview of smartphone applications designed for the self-management of low back pain. The study involved a comprehensive search of the existing literature and mobile platforms for relevant applications. Conducted by health professionals from “Tor Vergata” University, the research team has extensive experience in studies related to outcome measures in Italy. This review adheres to the 27-item PRISMA guidelines for reporting systematic reviews.

### 2.1. Inclusion and Exclusion Criteria

**Figure 1:**
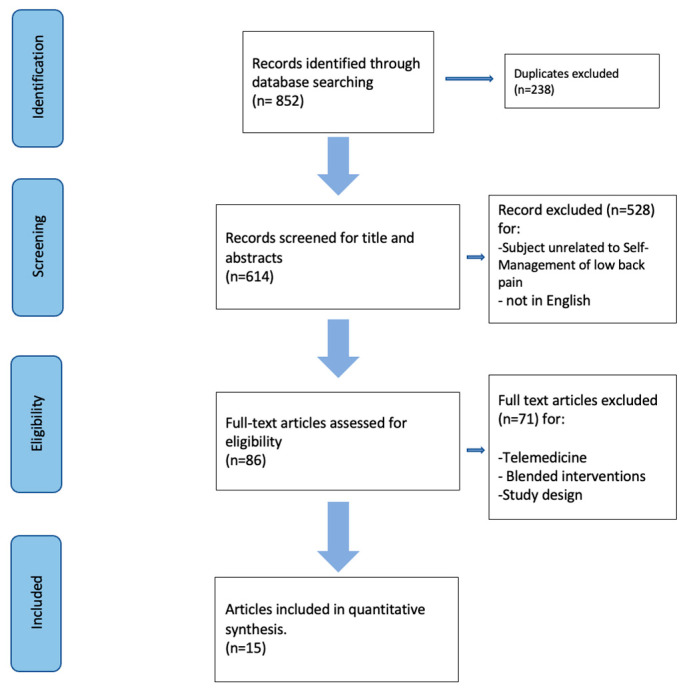
Process selection of the study.

Studies for this review were included according to the following criteria (Figure 1): types of studies, participants, and intervention. Studies were limited to people with low back pain (LBP), regardless of the clinical course or how long it had been since diagnosis. Studies with mixed diagnosis samples were included if a subgroup of participants with LBP could be identified and separate data were available.

All studies based on self-management using a smartphone app to relieve pain, improve function and promote physical well-being in individuals with low back pain were considered. The included papers described various LBP self-management interventions, such as exercises for motor control, strength or flexibility, pain management tips, mindfulness, or relaxation exercises.

There were no restrictions on study design or publication period. Only studies in English were considered.

The following were excluded: (a) studies related to telemedicine; (b) blended rehabilitation interventions (self-management combined with live sessions with healthcare professionals); and (c) study protocols.

### 2.2. Search Strategy

All the studies in the available literature that included the keywords (“low back pain”, “mobile applications”, “telemedicine”, “smartphone”) connected by the Boolean operator “AND” were considered. Appendix A shows the search queries strings used. Studies were selected for inclusion through individualized systematic searches of six electronic databases that were systematically searched in November 2022: PUBMED, SCOPUS, WEB OF SCIENCE, CYNAL, PSYCINFO, and PEDRO. All potential studies were selected by two reviewers.

### 2.3. Selection Process of Studies

The titles, abstracts, and keywords selected from the databases were independently screened by two physical therapists (LS and LB). After the initial screening, studies that did not meet the inclusion criteria were systematically excluded, while those that appeared relevant were identified. A final list of eligible studies was then compiled, with any disagreements resolved through consensus. The studies that met the criteria underwent a full-text review to confirm their inclusion. The online software “Rayyan” (version: 4406419348369) was used for the screening process.

### 2.4. Synthesis Method

Two reviewers independently extracted study characteristics using Microsoft Excel 2019 (Microsoft Inc., Redmond, WA, USA). The extracted data included the country of the study, study objectives, population characteristics, diagnosis, app name, app content, cost, developer information, treatment outcomes, and platform (iOS/Android). The included studies were then examined for similarities in participants, interventions, and results. In cases of heterogeneity among the studies, a narrative synthesis was provided.

### 2.5. APP Search Strategy

Mobile applications were searched in May 2022 on the Italian Apple App Store and Google Play Store using the keywords “Low back pain”, “Lumbago”, and “Lombalgia”. The following inclusion criteria were applied: (a) the app was designed for LBP self-management; (b) it targeted adult users; (c) it was available in Italian or English; (d) it was free or did not require in-app purchases for full functionality; (e) it was available for download from both the iOS and Google Play Stores; and (f) it had been updated within the last three years. Two reviewers based in Italy (L.S. and L.B.) conducted an initial screening of the mobile applications by title and description. Any discrepancies in app selection were resolved by a third reviewer (G.G.) to reach a consensus. Subsequently, the apps were downloaded and tested, with those not meeting the inclusion criteria being excluded.

After identifying the apps to be included, data were extracted and an evaluation was carried out using the Mobile App Rating Scale (MARS) by four reviewers (L.S., L.B., G.G., F.B.). The MARS consists of 23 questions designed to evaluate engagement, functionality, aesthetics, information, and subjective quality of mobile applications. Additionally, there are six final, app-specific questions that can be customized to reflect the target health behavior or functionality of the application or study; however, this section was not evaluated in this study. The total MARS score is the mean of the mean scores for each of the four subscales (engagement, functionality, aesthetics, and information), rated on a scale from zero to five. All reviewers received training in the use of MARS through a tutorial available on YouTube [10]. Following the tutorial, the reviewers independently tested a single app, leading to a final discussion to reach a consensus. Discrepancies were resolved by a third reviewer, and ambiguous MARS items were clarified to ensure full comprehension of the scale.

Finally, the inter-rater reliability of MARS and the degree of agreement among evaluators were assessed by calculating the intraclass correlation coefficient (ICC) score [11].

## 3. Results

### 3.1. Study Selection

A total of 852 articles were initially identified and screened for inclusion criteria using the specified search terms. Of these, 238 duplicates were removed, leaving 614 articles for screening. After reviewing the titles and abstracts, 582 studies were excluded. Subsequently, 71 studies were excluded due to an inappropriate research design or lack of relevance to the self-management topic. Ultimately, 15 studies were included in the quantitative synthesis [6,8,12,13,14,15,16,17,18,19,20,21,22,23,24]. The selection process is detailed in Figure 1.

### 3.2. Study Characteristics

At the end of the screening and selection process, sixteen studies were identified: six randomized controlled trials (RCTs), three systematic reviews, three systematic assessments, one scoping review, one observational study, and one non-randomized clinical trial. Of the six RCTs [12,13,14,15,16,17], four reported statistically significant reductions in pain levels favoring the mHealth app group compared to physiotherapy, web-based education, web-based email support, no training, or placebo groups. Conversely, two RCTs did not show statistically significant differences in pain levels between the mHealth app and control groups. The studies varied in how they reported participant ages, with the mean age ranging from 18 to 50 years. Two studies [12,19] did not report participant ages, and only one study [16] had a majority of female participants.

The study by Lo et al. [18] reported a statistically significant reduction in the Numeric Rating Scale (NRS) (*p* = 0.04) between pre- and post-treatment assessments. In the sole non-RCT study [19], there was an increase in the disability score (from 6.08 to 7.5; *p* = 0.01). No significant differences were found between groups or sessions in Pressure Pain Thresholds (PPTs).

Various smartphone apps were used in the intervention groups across the studies. The apps included “Relieve my back” [12], “Snap care” [13], “Truth about low back pain” [14], “ViViRA App” [15], “Kaia App” [16,17], “Well health” [18], and “BackFitApp” [19]. The app content was based on multidisciplinary interventions focusing on physical strengthening and stretching exercises for the lower back or core area [12,15,16,17], education and pain management advice [12,13,14,16,18], and mindfulness and relaxation techniques [16,17]. The outcome measurement tools for assessing pain and disability varied across the studies. Pain was measured using the NRS [11,15,17], NPRS [12,16], VNRS [14], PROMIS [13], and PPTs with an algometer [19]. Disability was assessed in three studies [12,13,19] using the Oswestry Disability Index (ODI) and Modified Oswestry Disability Index (MODI).

The remaining seven studies [6,8,20,21,22,23,24] were reviews aimed at evaluating papers discussing apps for self-management of low back pain [6,8,24], assessing apps qualitatively [20,22,23], or both [21]. Further details on these studies can be found in Table A1 and Table A2 (Appendix A). None of the applications from the studies were included in our evaluation as they were either not available in app stores or did not meet the inclusion criteria.

### 3.3. Overview of Apps Results

**Figure 2:**
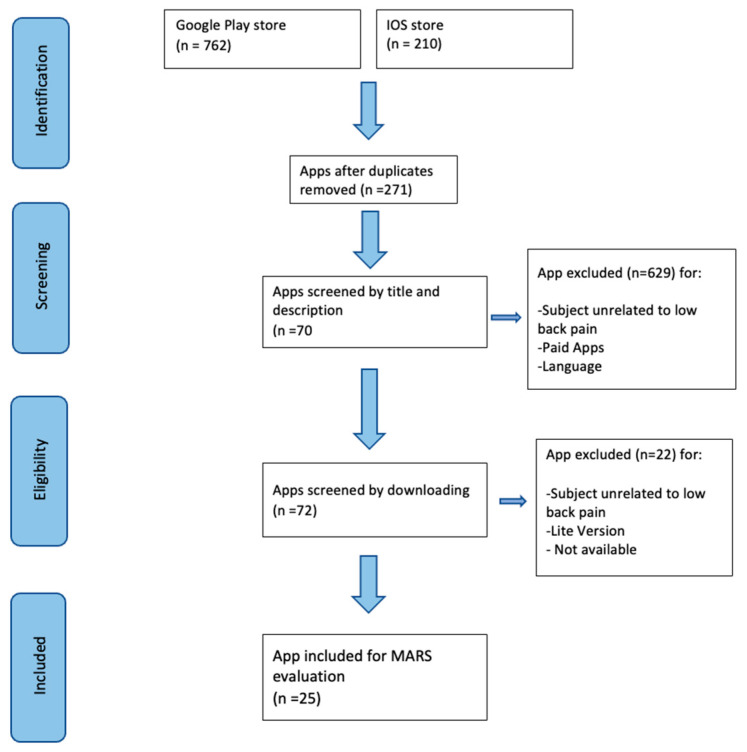
Process selection of the apps.

Our search yielded 762 items on the Play Store and 210 on iOS. Following the exclusion of 271 apps, screening was performed based on the app descriptions, resulting in 55 apps meeting the inclusion criteria for the Play Store and 17 for iOS. Subsequently, the apps were downloaded, and after a final screening, 20 apps from the Google Play Store and 5 from iOS were assessed using the MARS scale. The selection process is illustrated in Figure 2. All apps fell within the categories of health and well-being (iOS) and Health & Fitness (Google Play Store). Data extraction for the number of app downloads was only feasible for Android apps, as this information is not accessible on the Apple App Store. The app with the highest star rating on both platforms was “Atlas Low Back Pain”; however, not all apps had a sufficient number of ratings to display this score.

App downloads ranged from 100+ to 100,000+. The most downloaded applications from the Google Play Store were “Esercizi per il mal di schiena” developed by FitStar Apps s.r.o. (100,000+ rating of 4.6 out of 5), “Back Pain Relief Yoga at Home” developed by Dr. Zio–Yoga Teacher (100,000+ rating of 4.4 out of 5), and “Posture Correction Exercises” developed by Gym Fitness Technology (100,000+ rating of 3.7 out of 5). The mean overall MARS score obtained was 2.88 out of 5. The highest-rated app was “Pain Guru” by Dr. Giresh Kanji (4.09), while the lowest score was for “Lower Back Pain Exercises” by Steveloper (1.88); both apps were found in the Google Play Store. “Pain Guru” scored 4/5 in Engagement, 4.75 in Functionality, 4.5 in Aesthetics, and 3.14 in the Information sub-scale. In contrast, “Lower Back Pain Exercises” scored 1.8 in Engagement, 3.25 in Functionality, 1.33 in Aesthetics, and 1.14 in Information. “Pain Guru” aims to investigate the cause of low back pain, aid in diagnosis, and provide necessary information for pain management. Conversely, “Lower Back Pain Exercises” aims to offer a list of exercises beneficial for managing low back pain; however, its rudimentary graphics and lack of comprehensive information impede user engagement. Notably, there was no correlation observed between MARS scores and app ratings in the iOS and Google Play Stores. Detailed information regarding the apps evaluated using MARS is provided in Table A3 (Appendix A).

The Intraclass correlation coefficient (ICC) (2,1) calculated across all applications indicated good reliability values (ICC = 0.791, 95% C.I. [0.583, 0.901]). ICC estimates and their 95% confidence intervals (95% C.I.) were computed for 20 Play Store apps and 5 iOS apps using the SPSS statistical package version 29 (SPSS Inc., Chicago, IL, USA), based on a mean rating (k = 2), absolute agreement, two-way random effect model or ICC (2,1). ICC values were interpreted as follows: poor if smaller than 0.50, fair for values between 0.50 and 0.75, good between 0.75 and 0.90, and excellent above 0.90 (2,1).

## 4. Discussion

Currently, smartphone applications are increasingly employed in managing various health conditions, including chronic illnesses and post-surgical recovery, with several demonstrating efficacy in enhancing investigated outcomes and treatment adherence [25,26].

Notably, while not explicitly endorsed in recent guidelines [27,28], mobile applications hold promise in advancing the landscape of low back pain (LBP) rehabilitation when juxtaposed with alternative musculoskeletal modalities [29]. The diffuse presence of smartphones represents significant strides in the self-management of musculoskeletal disorders, in which these applications can operate autonomously or complementarily with live sessions to bolster treatment adherence. Noteworthy efforts have been made to assess the content quality of such applications, albeit predominantly within international contexts [20,22,23]. Our review is focused on assessing free mobile applications without in-app purchases to ensure their suitability for integration into clinical practice, particularly in resource-constrained settings where more expensive self-management systems may not be feasible. 

Among the primary studies analyzed, only the Kaia app is accessible to download for the general population on IOS or Play store in the Italian market. However, it was not evaluated through MARS because it was not free. 

For the purpose of assessment using the MARS scale, we identified 25 apps on the IOS and Play Stores that met our predetermined inclusion criteria. Overall, the analyzed apps demonstrate below-average quality (see Table A3). Many of these apps received the highest scores in the “functionality” subcategory (SM = 3.76). This suggests that most apps prioritize operational aspects without due consideration for features that could enhance user engagement and relevance to a broader user base. Conversely, the lowest scores were observed in the “information” subcategory. High-quality information within the app is crucial for ensuring safe usage. While it is imperative to investigate the effectiveness of interventions facilitated by each specific mobile app, such studies are rarely undertaken. In our evaluation, a majority of the apps employed information and education as their intervention strategy, followed by advice, strategies, and skills training. The predominant technical feature used to support these strategies was sending reminders. However, in many instances, the proposed interventions lacked clear references, raising questions about the reliability of app usage. In addition, evaluation through MARS showed that most of the apps incorporated only exercise programs, neglecting education to support knowledge and self-care strategies. Indeed, the use of low-quality information does not reflect what is stated in the most recent guidelines on the management of low back pain [28], limiting its dissemination and use in the management of these patients. Notably, no scientific evidence was found to substantiate the efficacy of any of the apps included in this study; rather, all apps appeared to be commercially driven. Lastly, none of the downloadable apps included in the study have ever been tested in clinical efficacy studies.

MARS sections on engagement and aesthetics highlight that most of the included apps pay little attention to aspects of design and user engagement; this can be a major barrier to app usage adherence.

The studies analyzed in our review show that app-based self-management strategies could be effective in reducing pain in low back pain patients; these findings align with previous reviews conducted on mHealth applications focused on pain management [20,21,26]. Ensuring the safety of apps intended for healthcare purposes remains a paramount concern for clinicians. Proper prescription and usage necessitate clear guidance on activities and exercises suitable for independent home-based practice versus those requiring supervised implementation. Ultimately, our study underscores that the quality of freely available apps in Italy generally falls below that of paid apps reported in other investigations [23].

### 4.1. Study Limitations

Firstly, it is important to acknowledge the potential for selection bias in the literature screening process of this review. Studies that did not explicitly mention a smartphone app-based intervention in the title or abstract were excluded. Additionally, our search focused solely on interventions utilizing smartphone apps in isolation, excluding those employing combined interventions, commonly referred to as “blended” approaches. Moreover, the generalizability of the findings may be limited due to several factors. Some studies with large sample sizes failed to report the duration or type of pain (acute, subacute, or chronic), which could impact the interpretation of results. Additionally, the absence of data regarding patients’ acceptance of mHealth apps for LBP self-management, as well as details regarding the intensity and frequency of app usage, further restricts the generalizability of our findings. Furthermore, it is worth noting a significant limitation in the assessment using MARS, as highlighted in a study on mHealth Apps conducted in the German language [30]. These apps are primarily judged based on acceptable or good quality, despite recent findings indicating otherwise [31]. A review revealed that the quality of self-management tools for low back pain (LBP) was notably lacking. Despite the high reliability reported [9], it is essential to acknowledge the inherent subjectivity in reviewer assessments, which may have influenced the assigned scores. Consequently, caution is warranted when interpreting the results outlined in this review. Furthermore, it is conceivable that the features of the applications discussed here may differ from those of updated versions available in app stores at the time of publication. This dynamic nature of application development inevitably introduces a degree of uncertainty.

Indeed, while the decision to exclusively evaluate free apps offers potential advantages for the practical application of clinical findings, it may inadvertently alter the presentation of results in this review. The great range of paid apps available today could either confirm or challenge the findings of this study. The discussion surrounding the cost-effectiveness of paid apps remains ambiguous, as noted in the Lewkowicz et al. investigation [32]. Conversely, free apps may represent an avenue to foster a culture of self-management, particularly in regions with developing healthcare infrastructures [33].

### 4.2. Future Study Recommendations

There is a clear need for larger scale RCTs examining the impact of smartphone apps on self-management of LBP, particularly with comparative treatments. Furthermore, the duration of interventions varies across studies, ranging from 6 to 12 weeks. As a result, drawing definitive conclusions regarding the long-term efficacy of mHealth apps for LBP self-management remains challenging, and their suitability for specific types of pain warrants further exploration. Thus, longer follow-up periods (>12 weeks) are essential to assess both the sustained effects and any clinically significant changes over time.

Given the current lack of evidence-based insights, increased support from scientific and academic institutions in the development of such mHealth solutions could encourage the development of higher quality and more clinically relevant apps. Moreover, based on our findings, we advocate for the formulation of guidelines to inform the development of new apps, with an emphasis on integrating clinical assessment and follow-up functionalities within the apps themselves.

## Appendix A

**Table A1:** Characteristics of the studies.

Sources, First Author, Year, Country	Study Design	Selection; *n* Women (%), Years	Interventions	App	Outcome for Pain and Disability	Effect on Pain and Disability	Platform
Almhdawi et al., 2020, Giordania [12]	RCT	Workers (office); Group 1 = 14 (34.1%) Group 2 = 8 (19.5%).	Tips and instructions, office stretching exercises and home strengthening exercises. The application sends reminders to do the exercises and maintain proper posture.	Relieve my back	NRS, ODI	Pain reduction between groups (*p* < 0.001). ODI score increased (*p* < 0.001).	Not reported
Chhabra et al., 2018, India [13]	RCT	Ambulatory patients. App group = 45 (-) Control group = 48 (-), 41 years old.	Motivate, promote, and guide participants to increase their level of physical activity and adherence to exercise.	Snapcare	NPRS, MODI	No statistically significant difference for pain outcome. Improvement in disability outcome.	Not reported
Rhon et al., 2021, USA [14]	RCT	Military population. Intervention Group = 103 (-) Control Group = 105 (-) Age: 18–50 aa.	A guided video-based education session	Truth About Low Back Pain	PROMIS	PROMIS subscales were significantly different between group.	Not reported
Weise et al., 2022, Germany [15]	RCT	Patients recruited through newspaper advertisement. 215 patients. intervention group = 108 (-) and control group = 107 (-). Age > 18 years.	Self-guided exercise program	ViViRA app	VNRS	Significant and clinically relevant reduction in pain outcome throughout the 12-week duration of the program.	IOS and Android
Toelle et al., 2019, Germany [16]	RCT	Patients recruited through Facebook advertisement. Interventions Group = 35 (72.9) Control group = 31 (67.4), mean age 41.	Multidisciplinary pain treatment involves three therapy modules: (1) back pain-specific education, (2) physiotherapy/physical exercise, and (3) mindfulness and relaxation techniques	Kaia App	NRS	There were no significant differences between the groups at baseline. At 12 weeks follow-up, the Kaia app group reported significantly lower pain intensity.	IOS and Android
Priebe et al.,2020, Germany [17]	RCT	Patients recruited through Facebook advertisement and by general practitioner. Intervention group: 933, Female (65), mean age 42 years. Control: 312, Female (64), mean age 37.	Multidisciplinary pain treatment involves three therapy modules: (1) back pain-specific education, (2) physiotherapy/physical exercise, and (3) mindfulness and relaxation techniques	Kaia App	NPRS	The Intervention group showed a significantly stronger pain reduction compared to the control group after 3 months. The intervention group was also superior in secondary outcomes.	IOS and Android
Lo et al., 2018, China [18]	Observational Study	General population; 39 (24%), 18 to 65 years of age.	Tailored exercise rehabilitation program and self-management advice based on their presenting symptoms, complying with the NICE guidelines and a pain education program	Well Health	NRS	Statistically significant decreased pain between pre- and post- (*p* = 0.04)	Google Play, iOS, Mi Store (UK, USA, China)
Sitges et al., 2022, Spain [19]	Nonrandomized clinical trial	General population, n = 57; self-managed group (n = 27), face to face group (n = 30).	Intervention consisted of the following: (1) viewing a pain education video <4 min in duration, (2) answering a question about the video to ensure that participants have watched it; and (3) performing an approximately 50 min exercise session	BackFitApp	PPTs, ODI	There was an increase in the disability score (6.08 vs. 7.5; *p* = 0.01). No significant differences between the groups or sessions were found in PPTs	IOS and Android

NRS = Numeric rating scale, NPRS = Numerical pain rating scale, VNRS = Verbal numerical rating scale, MODI = Modified Oswestry Disability Index, ODI = Oswestry Disability Index, PROMIS = Patient-Reported Outcomes Measurement Information System, PPTs = Pressure pain thresholds with algometer.

**Table A2:** Characteristics of the studies (review).

Sources, First Author, Year, Country	Study Design	Key Words/Populations	Criteria for Inclusion/Exclusion	App	Study Results	Platform
Didyk et al., 2021, Australia [6]	Systematic review	Search terms: combination with terms back pain and smartphone. included studies were randomized controlled trials	INCLUSION: Stand-alone applications, without the need for external devices, in English and available in Oceania and had to contain at least one self-managed active rehabilitation intervention recommended by NICE guidelines. EXCLUSION: Apps on prevention, diagnostic tests, risk factors, if they did not include an intervention or deal with monitoring, if they aimed to market or advertise products or health centers, if they provided only general information such as anatomy or risk factors, if they did not allow for managing interventions in a way that was tailored to the individual person.	Snapcare (Chhabra), Fitback program (Irvine), telerehabilitation-based McKenzie therapy app (Mbada), Kaia App (Priebe, Toelle), Pain Care App (Yang)	Three studies reported a significant decrease in pain intensity in the intervention group. One study reported no significant difference between groups in pain self-efficacy. One study reported a significant reduction in disability in the intervention group. Two studies reported no between-group differences in quality of life. One study reported no correlation between adherence (app use) and change in pain intensity and one study reported that app use mediated the effect of teleconsultations on pain improvements.	Not reported
Stark et al., 2022, Australia [8]	Systematic review	The search terms included [APP] AND [[Orthopedic] OR [Neurosurgery]]. Articles.	INCLUSION: All studies were included that presented their results in English, German, or French, analyzing the outcome of smartphone app-based rehabilitation in back pain patients and those following spine surgery. EXCLUDED: Non-accessible full articles, letters to the editors and comments were excluded, as well as those which failed to present functional outcome following rehabilitation.	Kaia App, Snapcare, Fitbit App, FitBack app. In the remaining studies, the app used was not specified.	The VAS score was presented in all studies. without significance between the interventional and control group. Only one research group found significantly higher improvement in PROMs. for the application group.	Not reported
Machado et al., 2016, Australia [21]	Systematic assessment	Search Terms: “low back pain”, “back pain”, and “lumbago”. Smartphone app	INCLUSION: App in English, available to general public, and were self-contained product. No limitations on the cost. created and updated during 2015-2017. Specifically for self-management. EXCLUSION: Apps focused on diagnostic and prevention. Were excluded app for lbp on pregnancy and sciatica.	61 app: 24 app store, 33 play store, 4 both stores.	The overall quality of these apps was low, since they lacked engaging features, presented unattractive layouts, and provided. questionable and low-quality information. None have been tested for effectiveness in reducing the symptoms of LBP.	24 App store, 33 play store
Rintala et al., 2019, Finlandia [24]	Scoping review	Search terms: (i.e., “smartphone app”, “technology”, “health app”, and “mobile health”), LBP (i.e., “low back pain”, “low back ache”, “back pain”, “lumbago”, “acute lower back pain”, and “chronic lower back pain”), and self-management (i.e., “self-management” and “self-care”). “back pain mobile self”, “back pain mhealth”, and “back pain smartphone” for PEDRO Database. Articles.	INCLUSION: Peer reviewed articles published between 1 January 2015, and 17 June 2021. Studies aimed to explore the use of mHealth apps for self-management in people with LB. Any type of interventional study, cohort study, or possible case series, including an interventional period for >1 participant. Studies published in English. EXCLUSION: MHealth apps did not include self-management content, did not include an interventional study period, or if participants had been diagnosed with pain other than LBP. Study protocols, opinion articles, and studies other than interventional, cohort, or case series	(1) FitBack. (2) Snapcare. (3) Kaia App. (4) Relieve My Back. (5) Kaia App. (6) Kaia App. (7) SelfBACK	A total of 5 different mHealth apps were identified, of which 4 contributed to a statistically significant reduction in LBP. and clinically meaningful changes.	Not reported
Didyk et al., 2022, Australia [22]	Systematic assessment	Search terms: low back pain, spinal pain. Smartphone App	INCLUSION: Free and paid apps that did not involve external devices, for self-management of lumbago available in English. Apps had to contain at least one self-management component mentioned in NICE guideline recommendations. EXCLUSION: prevention apps, diagnostic tests, linked to risk factors, pain monitoring, or had advertising purposes. Also excluded were apps containing only general information or that did not allow for tailored use by the user.	(1) TrackActive Me: Virtual Physio; (2) Injurymap—effective exercise therapy (3); The truth about low back pain; (4) Pocket spine doc; (5) Exercises for back, neck and posture moovbuddy; (6) Selfback—Guest; (7) The back pain App; (8) BacktrainerHD; (9) Regimen—back pain relief; (10) Lower back pain and sciatica relief exercises; (11) Lower back yoga—floor class; (12) Back pain—causes, symptoms, treatments; (13) Bella’s lower back pain app; (14) Back pain causes and treatment; (15) NHS 24 MSK help; (16) My back injury; (17) Lower back pain treatment—tips and knowledge; (18) Yoga poses for lower back pain relief; (19) Lower back pain; (20) Symmetry exercise for low back pain; (21) Back pain relief in 7 days—yoga, exercise and diet; (22) Back pain exercises; (23) Sciatica treatment; (24) Home remedies for sciatic neck pain; (25) Back pain relief.	The average quality of included apps was acceptable (mean MARS score of 3.9 out of a maximum possible 5). The potential for self-management and behavior change was low. No significant correlation between app consumer rating and MARS scores. Significantly correlation with app quality and price (*p* = 0.049).	(1, 3, 6, 8, 9, 11, 15, 16, 21) iOS; (2, 4, 13) Android e iOS; (5, 12, 14, 17, 18, 19, 21, 22, 23, 24, 25) Android
Escriche-Escuder et al., 2020, Spagna [23]	Systematic assesment	Search terms: Low back pain. Smartphone App	INCLUSION: low back pain-related apps that included feedback or interventions, also apps that included, among other conditions, low back pain. EXCLUSION: pain-themed apps (generically) in non-English and Spanish languages, with visualization problems, and paid. Apps with post-free download purchases were excluded.	(1) Back pain relief exercises; (2) Lower back yoga—floor class; (3) Regimen- back pain relief; (4) 6 Min Back Pain Relief; (5) Yoga Poses for Lower Back Pain Relief; (6) Lower Back Pain Exercises; (7) Escuela de Espalda; (8) WomenBackWorkout; (9) Healthy Spine and Straight Posture-Back exercises/Columna vertebral sana and Postura recta; (10) Back Doctor (FREE) Health, Stretch, Workout (11) Right Motion—Alivia tu dolor solo con ejercicios; (12) Healo; (13) Doado, Your Back Companion; (14) Bella’s Lower Back Pain Exercises; (15) Healure: Physiotherapy Exercise Plans; (16) Curable: Back Pain, Migraine and Chronic Pain Relief; (17) Improve Posture For A Healthy Spine	Apps generally have good overall quality, especially in terms of functionality and aesthetics.	(1, 2, 3) iOS; (4–17) Play Store
Carvalho et al., 2022, Brasile [20]	Systematic assessment	Search terms: Neck pain, Lumbago, Persistent neck and lower back pain, Spine pain, Nonspecific neck and lower back pain. Smartphone App	INCLUSION: Only apps that operate without additional supports (external devices), developed between 2019 and 2021, language in Brazilian Portuguese, and that included interventions about: education/ counseling/ exercise/ patient health monitoring. EXCLUSION: apps for commercial and advertising purposes.	(1) Exercícios para dores nas cost (2) Exercícios para Coluna (3) MoovBuddy- Exercícios Físicos (4) Saúde da Coluna-Fisioterapia (5) Coluna vertebral sana, Postura and Costas treino (6) Exercícios para dor no pescoço—workout em casa (7) Exercícios para dores nas costas Fitness -Coach (8) Meu pescoço (9) Movimento Certo—Cuide sua dor so com exercícios (10) Treino para abdominais, core e costas em casa	Using MARS, the apps scored poorly in terms of quality, with an overall mean score ± standard deviation of 2.75 ± 0.63 on a scale of 1–5 points.	(1, 2, 3, 4) iOS e Android (5, 6, 7, 8, 9, 10) Android

**Table A3:** Characteristics of the Apps.

App Name	Author	Categories	Platform	Rating	Download	Intervention	Engagement Mean Score =	Functionality Mean Score =	Aesthetics Mean Score =	Information Mean Score =	MARS Total Score =
Esercizi per il mal di schiena	Vladimir Ratsev, NA	health and well-being	IOS	4.6 su 5	/	therapeutic exercise	2.9	4.37	4	2.21	3.37
Atlas low back pain	Atlas Health group Inc., NA	health and well-being	IOS	5.0 su 5	/	therapeutic exercise + education	3	4,12	3.83	2.78	3.44
Lower back Challenge Workout	Mobway solutions SRL, NA	health and well-being	IOS	/	/	therapeutic exercise	2	3.50	2	1.71	2.30
Lombalgia esercizi da fare	Stefan Roobol, NA	health and well-being	IOS	/	/	therapeutic exercise	3.6	3.87	4	2.64	3.53
BackBetter	Michael Simon Baliey, NA	health and well-being	IOS	/	/	therapeutic exercise + Midfulness	3.7	4.5	4.5	2.07	3.69
Esercizi di dolore Sciatica	tbeapps, Ankara, Turkey	Health & Fitness	Google Play store	/	+10,000	therapeutic exercise	1.20	4.12	1.49	1.35	2.04
Back Pain Relief Exercise Home	Gym fitness relief exercise Home, Bogotà, Colombia	Health & Fitness	Google Play store	/	+10,000	therapeutic exercise	2.2	3.62	2.5	1.57	2.47
Back Pain Relief Exercises	World Gym Fitness JS, NA	Health & Fitness	Google Play store	/	+10,000	therapeutic exercise	1.7	3.75	3	1.92	2.59
Esercizi per il mal di schiena	FitStar Apps s.r.o., Klatovy, Czechia	Health & Fitness	Google Play store	4.6	+100,000	therapeutic exercise	2.80	4.25	3.5	2.14	3.17
Lower back pain yoga	DhadbadatiApps, NA	Health & Fitness	Google Play store	/	+10,000	therapeutic exercise	1.7	4.12	1.5	1.85	2.17
Back Pain Relief Exercises	1B Studio Ltd., NA	Health & Fitness	Google Play store	/	+10,000	therapeutic exercise	3.2	3.87	3.16	2.28	3.13
Painguru	Dr Giresh Kanji, New Zeland	Health & Fitness	Google Play store	/	+100	Education	4	4.75	4.5	3.14	4.09
lower back pain exercises	1B Studio Ltd, NA.	Health & Fitness	Google Play store	/	+50,000	therapeutic exercise	3.40	3.75	3.5	2.85	3.25
Esercizi di terapia fisica	tbeapps, Ankara, Turkey	Health & Fitness	Google Play store	/	+10,000	therapeutic exercise	1.4	3.25	1.66	1.42	1.93
Esercizi dolore alla sciatica	Adminapps, Istanbul, Turkey	Health & Fitness	Google Play store	3.0	+10,000	therapeutic exercise	1.4	4	1.66	1.35	4.21
Esercizi di fisioterapia	Adminapps, Istanbul, Turkey	Health & Fitness	Google Play store	/	+10,000	therapeutic exercise	1.9	2.87	1.66	1.71	2.04
Back Pain Exercises and relief	ZE APK, NA	Health & Fitness	Google Play store	/	+100	therapeutic exercise	2.3	3.62	2.67	2.14	2.68
Back Pain Relief Yoga at Home	Dr.Zio—Yoga Teacher, NA	Health & Fitness	Google Play store	4.4	+100,000	therapeutic exercise	4.2	4.25	4.5	2.78	3.93
La lombalgia esercizi	Tbeapps, Ankara, Turkey	Health & Fitness	Google Play store	/	+50,000	therapeutic exercise	2.5	.37	2.33	2.21	2.64
Posture Correction Exercises	Gym Fitness Technology, NA	Health & Fitness	Google Play store	3.7	+100,000	therapeutic exercise	3.5	4.12	3.83	2.85	3.58
Lower Back Pain Exercises	Steveloper, Papendrecht, Netherlands	Health & Fitness	Google Play store	/	+10,000	therapeutic exercise	1.8	3.25	1.33	1.14	1.88
Back Pain App—Exercises, Help, Relief and Tips	21st Apps, NA	Health & Fitness	Google Play store	3.8	+10,000	therapeutic exercise	1.7	3.12	2.5	1.85	2.29
Esercizi per la lombalgia	Admineapps, Istanbul, Turkey	Salute e fitness	Google Play store	/	+10,000	therapeutic exercise	2.2	3.75	2.83	2.5	2.82
Physiotherapy Exercise Guide	Alpesh Patel, NA	Salute e fitness	Google Play store	/	+1000	therapeutic exercise	1.9	2.5	1.83	1.64	1.97
The back pain app	The Foundation PTS, NA	Salute e Fitness	Google Play store	/	+500	therapeutic exercise + education	2.4	3.5	2.66	2.78	2.84
